# Sonographic Renal Parenchymal Measurements for the Evaluation and Management of Ureteropelvic Junction Obstruction in Children

**DOI:** 10.3389/fped.2016.00042

**Published:** 2016-05-06

**Authors:** Jeremy C. Kelley, Jeffrey T. White, Jessica T. Goetz, Elena Romero, Jeffrey A. Leslie, Juan C. Prieto

**Affiliations:** ^1^University of Texas Health Science Center at San Antonio, San Antonio, TX, USA; ^2^San Antonio Military Medical Center, San Antonio, TX, USA; ^3^Driscoll Children’s Hospital, Corpus Christi, TX, USA

**Keywords:** urinary tract dilation, hydronephrosis, renal ultrasound measurements, UPJ obstruction, pyeloplasty

## Abstract

**Purpose:**

To correlate sonographic renal parenchymal measurements among patients with ureteropelvic junction obstruction (UPJO) labeled society of fetal urology (SFU) hydronephrosis grades 1–4 and to examine whether sonographic renal parenchymal measurements could be used to differentiate conservative vs. surgical management.

**Materials and methods:**

Retrospective chart review and sonographic renal parenchymal measurements (renal length, medullary pyramid thickness, and renal parenchymal thickness) were performed in patients with SFU grades 1–4 hydronephrosis secondary to UPJO managed between 2009 and 2014. Exclusion criteria included other concomitant genitourinary pathology or incomplete follow-up. Anterior–posterior renal pelvic diameter (APRPD) and radionuclide renography were also evaluated when available.

**Results:**

One hundred four patients with UPJO underwent 244 renal and bladder ultrasound (1,464 sonographic renal parenchymal measurements in 488 kidneys). Medullary pyramid thickness and renal parenchymal thickness progressively decreased from SFU grades 1–4 (*p* < 0.05). A similar trend was appreciated when comparing SFU grades 1 and 2 vs. 3 and 4, as well as SFU grades 3 vs. 4 (*p* < 0.05). SFU grade 3 and 4 patients who underwent pyeloplasty had longer renal length in comparison to those who were managed conservatively (*p* < 0.02).

**Conclusion:**

This is the first study that evaluates these objective, quantifiable sonographic renal parenchymal measurements in children with unilateral UPJO. These sonographic renal parenchymal measurements correlate closely with worsening of hydronephrosis graded by the SFU and APRPD classification systems. Prospective studies are needed to elucidate the role of sonographic renal parenchymal measurements in the management of children with UPJO.

## Introduction

Ureteropelvic junction obstruction (UPJO) is one of the most common causes of prenatal urinary tract dilation (UTD) in children accounting for up to 30% of cases (1). Currently, standard work-up for postnatal UTD secondary to UPJO includes renal and bladder ultrasound (RBUS), voiding cystourethrogram (or radionuclide cystography), and radionuclide renography. Serial RBUS and sometimes repeat functional scans are used to follow these children conservatively and determine the need for surgical management. The most widely accepted sonographic measurement systems to assess UTD are the semi-quantitative society of fetal urology (SFU) hydronephrosis grading system (2) and the quantitative anterior–posterior renal pelvic diameter (APRPD) (3–5). These grading systems do not demonstrate ideal inter-observer reproducibility (1).

The search for alternate methods of evaluation and follow-up of postnatal UTD has been ongoing. Significant research efforts have been put on RBUS to avoid the costs, radiation exposure, and invasiveness of radionuclide renography, CT scan, or MRI. A novel approach to evaluate the kidney using sonographic renal parenchymal measurements was presented by Kadioglu in 2010, who measured normal sonographic renal parenchymal measurements in healthy children (6).

First, the aim of our study was to evaluate these three sonographic renal parenchymal measurements in the settings of unilateral UPJO and to compare them with the APRPD and SFU grading systems. Second, we looked at those patients with SFU grades 3 and 4 who were managed medically vs. surgically to see if there was a difference in sonographic renal parenchymal measurements between the two groups.

## Materials and Methods

A multi-institutional retrospective study was conducted between 2009 and 2014 using medical records and images obtained from three children’s hospitals in South Texas (Driscoll Children’s Hospital, Methodist Children’s Hospital, and Children’s Hospital of San Antonio). Following IRB approval (study #11.018 and protocol #HSC20140444E) at these institutions, we identified patients with postnatal UTD secondary to UPJO using ICD-9 and CPT-codes. Only those children with unilateral UPJO without concomitant genitourinary pathology were included in the study. Examples of exclusion pathology include vesicoureteral reflux (VUR), bilateral UPJO, history of posterior urethral valves, duplicated collecting system, etc. Patients with incomplete imaging and/or follow-up were also excluded from the study.

**Figure 1:**
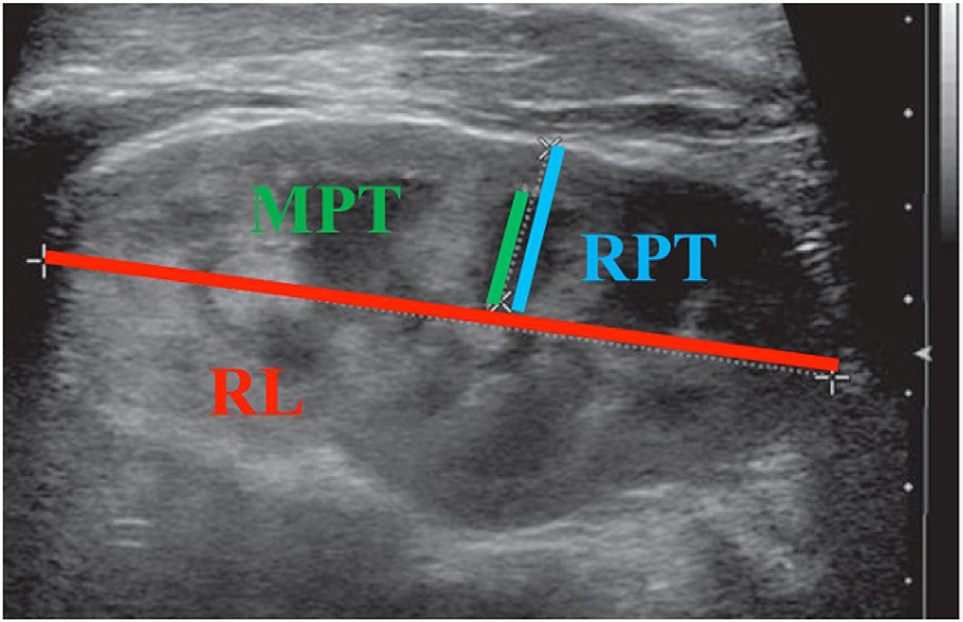
**Illustration of each sonographic renal parenchymal measurement**. MPT, medullary pyramid thickness; RPT, renal parenchymal thickness; RL, renal length.

As per hospital protocol, all patients undergoing RBUS were well hydrated orally with full bladders at the time of the study. Three sonographic renal parenchymal measurements were obtained for each kidney. Figure 1 illustrates how each measurement was obtained. Renal length was measured from the uppermost edge of the upper pole to the lowest edge of the lower pole. Medullary pyramid thickness is the distance from apex of the renal pyramid to its base. Renal parenchymal thickness is the distance between renal capsule and the apex of the pyramid. All measurements were taken in the sagittal plane using the image depicting the greatest length of the kidney. Medullary pyramid thickness and renal parenchymal thickness were measured in the middle third of the kidney; APRPD was measured in the transverse view for each kidney. All measurements were saved in digital files and reviewed by a single pediatric urologist (Juan C. Prieto) on two different occasions during the study period to improve intra-observer reliability.

Initial conservative management was given to all SFU grades 1–4 hydronephrosis except for those with grades 3 and 4 with a differential renal function ≤40%. Standard follow-up for SFU grades 3 and 4 included a RBUS every 3 months during the first year of life and every 4 months in the second year with frequency in subsequent years determined on an individual basis. Renal scans were ordered in patients with SFU grades 3 and 4 hydronephrosis according to the discretion of the clinician. Indications for pyeloplasty included worsening hydronephrosis, differential renal function decreased by ≥10%, and symptoms (e.g., UTI, vomiting, and failure to thrive).

A mixed linear regression model controlled for age and laterality was used to compare the sonographic renal parenchymal measurements with the APRPD and SFU grading systems to determine if there was a statistically significant difference in these sonographic renal parenchymal measurements with worsening degrees of hydronephrosis. A mixed model estimate of the surgical effect was used to test the association between treatment (surgical vs. conservative) and sonographic renal parenchymal measurements. The association tests between demographic and clinical measures were performed with non-parametric tests (Fisher’s and Kruskal–Wallis tests).

## Results

During the study period, 507 children were identified to have postnatal UTD. Patients with other causes of UTD and those with UPJO who did not meet the aforementioned inclusion criteria were excluded from the study. A total of 104 patients with unilateral UPJO underwent a total of 244 RBUS with 488 kidneys being measured. This yielded a total of 1,464 sonographic renal parenchymal measurements to be included in our analysis. Additionally, APRPD was measured in 231 RBUS (462 kidneys). Fifty-nine patients were managed conservatively, and 45 underwent pyeloplasty. Table 1 shows the baseline demographic data, and Table 2 shows the mean and Standard Deviation (SD) of each sonographic renal parenchymal measurement.

**Table 1:** **Baseline demographic data**.

Variable	All patients (%)
Age	
Mean ± SD	2.44 ± 4.18
Gender	
Male	79 (76.96)
Female	25 (24.04)
Laterality	
Left	77 (74.04)
Right	27 (25.96)
SFU	
0	0 (0)
1	25 (24.04)
2	21 (20.19)
3	23 (22.12)
4	35 (33.65)
Treatment	
Conservative	59 (56.73)
Surgery	45 (43.27)

**Table 2:** **Mean and SD of all sonographic renal parenchymal measurements (independent of age and laterality)**.

SFU	*n*	MPT (mm)	RPT (mm)	RL (mm)	APRPD (mm)
0	172	6.06 ± 1.99	11.04 ± 3.23	67.32 + 17.73	0.62 ± 2.83
1	86	5.86 ± 1.96	10.35 ± 3.1	63.13 ± 13.94	5.78 ± 6.88
2	71	5.05 ± 2.85	9.54 ± 3.97	68.19 ± 16.62	9.01 ± 8.36
3	65	3.41 ± 1.95	9.1 ± 3.79	78.91 ± 22.83	17.95 ± 13.08
4	94	1.81 ± 1.42	5.41 ± 2.42	81.51 ± 25.1	25.22 ± 15.39

*MPT, medullary pyramid thickness; RPT, renal parenchymal thickness; RL, renal length; APRDP, anterior–posterior renal pelvis diameter; SFU, society of fetal urology*.

**Figure 2:**
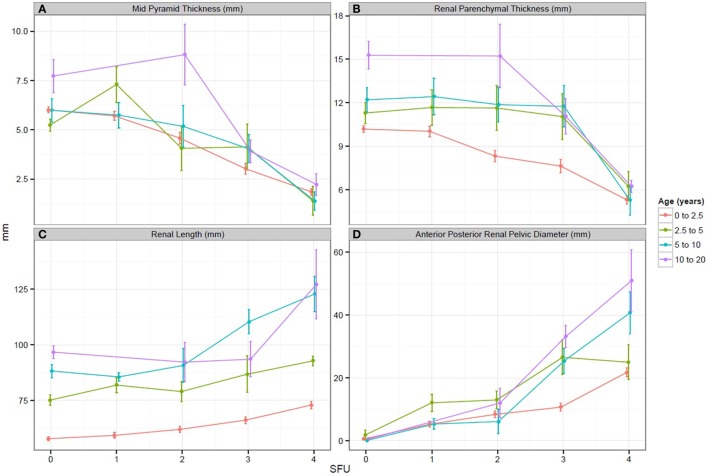
**(A–D)** Graphs representing the change in each sonographic renal parenchymal measurements with worsening hydronephrosis. SFU, society of fetal urology hydronephrosis grade.

In regards to our study goals, when comparing all SFU grades, both medullary pyramid thickness and renal parenchymal thickness demonstrated a significant decrease with worsening hydronephrosis (*p* < 0.05 for both measurements). Similarly, when SFU grades 1 and 2 were compared to SFU grades 3 and 4, a statistically significant decrease for each measurement was also obtained (*p* < 0.05, Figures 2A,B). In comparing renal length to SFU grades, an increase in renal length correlated with worsening hydronephrosis (*p* = 0.06). Moreover, when SFU grades 1 and 2 were compared to grades 3 and 4, a significant increase in renal length was documented in the SFU 3 and 4 group (*p* < 0.05, Figure 2C).

When APRPD was compared to all SFU grades, an increase in APRPD was seen with worsening hydronephrosis (*p* < 0.05). Likewise, when SFU grades 1 and 2 were compared to SFU grades 3 and 4, a significant increase in APRPD was found in the SFU 3 and 4 group (*p* < 0.05, Figure 2D). All three sonographic renal parenchymal measurements were also compared to APRPD, and the same trend was appreciated. Medullary pyramid thickness and renal parenchymal thickness showed moderate negative correlations to increasing APRPD, *r* = −0.41 and −0.28, respectively. Renal length showed moderate positive correlation (*r* = 0.5).

In comparing those patients with SFU grades 3 and 4 who were treated with expectant vs. surgical management, only renal length showed a statistically significant difference (*p* = 0.02). Radionuclide renography was obtained in 23 patients; however, differential renal functions or washout data were not included in the evaluation, since the majority of the images were not available for review. On average, patients treated surgically had a renal length 10.51 mm (±4.436 mm) greater than those treated conservatively. Medullary pyramid thickness showed a trend toward decreasing renal length (0.625 ± 0.355 mm) but this measurement did not reach statistical significance (*p* = 0.08). It should be noted that this last finding is limited by the retrospective nature of our study resulting in the lack of data regarding the decision to perform surgery on individual patients. These findings are summarized in Table 3.

**Table 3:** **Differences in sonographic renal parenchymal measurements in those patients with SFU 3 or 4 hydronephrosis treated surgically vs. conservatively**.

Measure	Surgery vs. conservative (SE)	*p*-Value
Renal length (mm)	10.511 ± 4.436	0.0207
Mid pyramid thickness (mm)	−0.625 ± 0.355	0.0834
Renal parenchymal thickness (mm)	0.629 ± 0.646	0.3336
Anterior–posterior renal pelvis diameter (mm)	2.157 ± 2.691	0.4258

## Discussion

In the settings of UPJO in children, significant attention has been given to assessment of renal pelvis dilation, calyceal dilation, and qualification of the renal parenchyma; however, little attention has been given to objective quantification of the renal parenchyma. Evaluation of the renal parenchyma and renal growth is paramount, since it provides an estimate of the renal functional reserve. Two recognized classifications systems have been proposed to rank prenatal and postnatal UTD: that of Grignon et al. which relies on APRPD (7), while that of SFU relies on the extent of intrarenal calyceal dilatation (2). Additionally, others have attempted to integrate these systems into a semi-quantitative grading system. Onen proposed combining the degree of pelvicalyceal dilation with the percentage of loss of renal parenchyma (3–5). This alternate system does not offer an objective quantification of the renal parenchyma and has decreased inter-rater reliability in comparison to the SFU grading system (8).

Several other sonographic parameters have been studied to evaluate UTD in children: pelvicalyceal area, parenchymal to pelvicalyceal area (hydronephrosis index), calyx to parenchymal ratio, and pelvicalyceal volume on 3D RBUS (9–11). These measurements are difficult to assess and require access to specialized software or equipment, making them less commonly used in clinical practice (1).

In patients with VUR, Shortliffe et al. have extensively studied renal volume measurements estimated from CT and MRI. They showed that renal volume could estimate differential function; renal volume is decreased in refluxing vs. non-refluxing kidneys (12). They also studied 2-D renal parenchyma area (RPA) from RBUS as a surrogate for renal volume. RPA was found to be smaller in children with high grade VUR (grades 4 and 5) in comparison to low grade VUR (grades 0–3) (13, 14).

As mentioned above, measurements of the renal parenchyma are complex to assess and to quantify. Due to these drawbacks, the pediatric urology community has not adopted renal parenchymal measurements in the evaluation and follow-up of congenital UPJO. Despite significant efforts made by the Multidisciplinary Consensus on the Classification of Prenatal and Postnatal UTD, renal parenchymal thickness remains a subjective categorization within this new classification system (1).

In an effort to find an objective quantifiable measurement of the renal parenchyma for the evaluation of UTD, our group introduced a recently described sonographic renal parenchymal measurements system for the postnatal evaluation of unilateral prenatal UPJO. This 2-D sonographic measuring system has the advantage of being easily reproduced and quickly obtained. We compared these three sonographic renal parenchymal measurements with the already established APRPD and SFU grading systems. We demonstrated a significant correlation of all sonographic renal parenchymal measurements with worsening hydronephrosis. A significant decrease in medullary pyramid thickness and renal parenchymal thickness was observed with worsening hydronephrosis (by the SFU grading system and APRPD). Furthermore, when comparing mild grades of hydronephrosis (SFU 1 and 2) with more severe grades (SFU 3 and 4), a significant increase in renal length was documented in the severe group (*p* < 0.05). To the best of our knowledge, this is the first study to use these quantifiable sonographic measurements of the renal parenchyma in the evaluation of postnatal UTD secondary to UPJO. Previous studies included subjective qualification and quantification of the renal parenchymal thickness but none used an objective quantifiable measurement system to correlate it with the most commonly used hydronephrosis grading systems.

Due to the retrospective nature of this study, we were not able to correlate our sonographic renal parenchymal measurements with the need for pyeloplasty. Even in this small cohort of surgeons, indications for the procedure were ill defined, and nuclear renal scans were not consistently available for review. However, we were able to demonstrate increased renal length and decreased medullary pyramid thickness in patients with SFU grades 3 and 4 hydronephrosis who underwent pyeloplasty.

The utility of radionuclide renography, SFU grading, APRPD, and other qualitative parenchymal thickness measurements has been studied as potential indicators for pyeloplasty. A report from Great Ormand Street indicated that pyeloplasty was only needed in approximately 25% of hydronephrotic kidneys when based on decreasing renal function, UTI, or pain (15). Subsequently, Koff and Campbell reported non-surgical management in 45 patients with SFU grades 2–4, finding none experienced decreasing renal function requiring surgery. Complete resolution of UTD was only seen in 2 out of 15 of the SFU grade 4 kidneys (16). Most recently, Sharifian et al. showed that hydronephrosis on postnatal ultrasounds correlated with antenatal diagnosis in 70% of patients. Furthermore, they showed that APRPD of 15 mm differentiated the surgical group with 95.2% sensitivity and 73.5% specificity (9). Their results were similar to that of Bouzada and colleagues (10). Dias et al. showed that the combination of prenatal and postnatal APRPD on postnatal ultrasound had a better accuracy in predicting need for pyeloplasty than SFU grading system (11). By combining fetal APRPD >18 mm with postnatal APRPD >16 mm, they predicted surgical status with 100% sensitivity and 86% specificity (11). Importantly, only three of the patients (8%) managed expectantly presented with UTI and no patients with acceptable ultrasound criteria later underwent pyeloplasty. Other measurements, such as calyceal depth-to-parenchymal thickness ratio, have been used to assess ultrasound changes post-pyeloplasty, but not to predict surgical intervention (17).

To improve the sensitivity and specificity of the above measurements, others have integrated these variables into comprehensive quantitative models. Wang et al. performed a multivariate logistic regression model using the variables of unilateral or bilateral hydronephrosis, APRPD, and renal parenchymal volume to predict surgery with an accuracy of 78.77%, sensitivity of 81.63%, and specificity of 77.69% (18). Zhan et al. derived an ultrasound score, the sum of standardized grading system of APRPD, renal parenchymal thickness, and SFU grade (19). The authors found that a score of 6 or higher diagnosed pathologic hydronephrosis with a sensitivity of 89.8% and specificity of 94.2%. Cost et al. discovered that renal length parenchymal area is more accurate in estimating renal size and function than solitary sonographic renal parenchymal measurements in hydronephrotic kidneys (20). The ratio of renal longitudinal parenchymal area to pelvic area predicted surgical management for a cutoff of 1.6 with a sensitivity of 100% and specificity of 100%. However, obtaining this measurement is time consuming and requires significant computing resources that are not available to all practitioners.

We identified several drawbacks to our study. First, it is a retrospective study performed in three different institutions; this presents unique problems, such as different hydration protocols at these institutions, which will affect RBUS results. Second, due to the 2-D nature of the study using different sonogram machines, significant variation in intra- and inter-observer reliability was expected. To avoid inter-rater variability and to favor intra-observer reliability, one pediatric urologist performed all measurements in two separate occasions during the study period.

Prospective studies are needed to define range values of these 2-D sonographic renal parenchymal measurements for the prenatal and postnatal UDT classification system. Furthermore, it will be necessary to elucidate the value of these measurements in the postnatal management of UPJO by correlating these sonographic renal parenchymal measurements with worsening hydronephrosis and decrease differential renal function. Due to the simplicity and reproducibility of these measurements, there is a significant potential for these measurements to become useful practical tools for pediatric urologists when making the decision between conservative vs. surgical management in children with UPJO.

## Conclusion

Although there are many methods of grading UTD, any clinically useful technique should be easily reproducible, predictive of renal damage, and useful for assessing worsening or improvement of urinary obstruction. Here, we present the first study that objectively evaluates these 2-D sonographic renal parenchymal measurements in patients with UTD secondary to prenatal UPJO. These sonographic renal parenchymal measurements correlate closely with worsening hydronephrosis, as assessed by SFU and APRPD grading systems. Prospective studies are needed to elucidate the value of sonographic renal parenchymal measurements as an objective quantifiable tool to evaluate and follow children with UPJO and to determine if these measurements could serve as an additional indication for surgical intervention.

## Author Contributions

The above listed authors (JK, JW, JG, ER, JL, and JP) were involved in the design of the submitted study. They were involved in the acquisition, analysis, and interpretation of the data. Each author was involved in drafting the manuscript and editing it for accuracy and content. Each author was included in approving the manuscript for final approval before submission. Additionally, all authors agreed to be responsible for all aspects of the submitted work.

## Conflict of Interest Statement

The authors declare that the research was conducted in the absence of any commercial or financial relationships that could be construed as a potential conflict of interest.

## Abbreviations

APRPD, anterior–posterior renal pelvis diameter; SFU, society for fetal urology; UTD, urinary tract dilation; VUR, vesicoureteral reflux.

## References

[1] Nguyen HT Herndon CD Cooper C Gatti J Kirsch A Kokorowski P The Society for Fetal Urology consensus statement on the evaluation and management of antenatal hydronephrosis. J Pediatr Urol (2010) 6:212. 10.1016/j.jpurol.2010.02.205 20399145

[2] Fernbach SK Maizels M Conway JJ. Ultrasound grading of hydronephrosis: introduction to the system used by the Society for Fetal Urology. Pediatr Radiol (1993) 23:478. 10.1007/BF02012459 8255658

[3] Onen A. An alternative grading system to refine the criteria for severity of hydronephrosis and optimal treatment guidelines in neonates with primary UPJ-type hydronephrosis. J Pediatr Urol (2007) 3:200. 10.1016/j.jpurol.2006.08.002 18947735

[4] Onen A Jayanthi VR Koff SA. Long-term followup of prenatally detected severe bilateral newborn hydronephrosis initially managed nonoperatively. J Urol (2002) 168:1118. 10.1097/00005392-200209000-00067 12187248

[5] Onen A. Treatment and outcome of prenatally detected newborn hydronephrosis. J Pediatr Urol (2007) 3:469. 10.1016/j.jpurol.2007.05.002 18947797

[6] Kadioglu A. Renal measurements, including length, parenchymal thickness, and medullary pyramid thickness, in healthy children: what are the normative ultrasound values? AJR Am J Roentgenol (2010) 194:509. 10.2214/AJR.09.2986 20093617

[7] Grignon A Filion R Filiatrault D Robitaille P Homsy Y Boutin H Urinary tract dilatation in utero: classification and clinical applications. Radiology (1986) 160:645. 10.1148/radiology.160.3.3526402 3526402

[8] Kim SY Kim MJ Yoon CS Lee MS Han KH Lee MJ. Comparison of the reliability of two hydronephrosis grading systems: the Society for Foetal Urology grading system vs. the Onen grading system. Clin Radiol (2013) 68:e484. 10.1016/j.crad.2013.03.023 23684519

[9] Sharifian M Esfandiar N Mohkam M Dalirani R Baban Taher E Akhlaghi A. Diagnostic accuracy of renal pelvic dilatation in determining outcome of congenital hydronephrosis. Iran J Kidney Dis (2014) 8:26. 10.1016/j.juro.2013.02.014 24413717

[10] Bouzada MC Oliveira EA Pereira AK Leite HV Rodrigues AM Fagundes LA Diagnostic accuracy of fetal renal pelvis anteroposterior diameter as a predictor of uropathy: a prospective study. Ultrasound Obstet Gynecol (2004) 24:745. 10.1002/uog.1764 15586376

[11] Dias CS Silva JM Pereira AK Marino VS Silva LA Coelho AM Diagnostic accuracy of renal pelvic dilatation for detecting surgically managed ureteropelvic junction obstruction. J Urol (2013) 190:661. 10.1016/j.juro.2013.02.014 23416643

[12] Chang SL Caruso TJ Shortliffe LD Magnetic resonance imaging detected renal volume reduction in refluxing and non-refluxing kidneys. J Urol (2007) 178:2550 10.1016/j.juro.2007.08.053 17937957

[13] Wong IY Copp HL Clark CJ Wu HY Shortliffe LD. Quantitative ultrasound renal parenchymal area correlates with renal volume and identifies reflux nephropathy. J Urol (2009) 182:1683. 10.1016/j.juro.2009.03.075 19692072

[14] Pruthi RS Angell SK Dubocq F Merguerian PA Shortliffe LD. The use of renal parenchymal area in children with high grade vesicoureteral reflux. J Urol (1997) 158:1232. 10.1097/00005392-199709000-00146 9258182

[15] Dhillon HK Prenatally diagnosed hydronephrosis: the Great Ormond Street experience. Br J Urol (1998) 81(Suppl 2):39 10.1046/j.1464-410X.1998.0810s2039.x 9602794

[16] Koff SA Campbell K. Nonoperative management of unilateral neonatal hydronephrosis. J Urol (1992) 148:525. 1640515 10.1016/s0022-5347(17)36644-2

[17] Imaji R Dewan PA. Calyx to parenchyma ratio in pelvi-ureteric junction obstruction. BJU Int (2002) 89:73. 10.1046/j.1464-410X.2002.02543.x 11849165

[18] Wang J Ying W Tang D Yang L Liu D Liu Y Prognostic value of three-dimensional ultrasound for fetal hydronephrosis. Exp Ther Med (2015) 9:766. 10.3892/etm.2015.2168 25667626 PMC4316903

[19] Zhan X Tao G Cheng L Liu F Li H Liu S. Ultrasound score: a new method to differentiate fetal physiological and pathological hydronephrosis. Eur J Obstet Gynecol Reprod Biol (2010) 151:26. 10.1016/j.ejogrb.2010.02.046 20395034

[20] Cost GA Merguerian PA Cheerasarn SP Shortliffe LM Sonographic renal parenchymal and pelvicaliceal areas: new quantitative parameters for renal sonographic follow-up. J Urol (1996) 156:725 10.1097/00005392-199608001-00045 8683769

